# ﻿A remarkable new species of *Paraparatrechina* Donisthorpe (1947) (Hymenoptera, Formicidae, Formicinae) from the Eastern Himalayas, India

**DOI:** 10.3897/zookeys.1203.114168

**Published:** 2024-05-30

**Authors:** Ramakrishnaiah Sahanashree, Aswaj Punnath, Dharma Rajan Priyadarsanan

**Affiliations:** 1 Ashoka Trust for Research in Ecology and the Environment, Royal Enclave, Srirampura, Jakkur Post, Bengaluru – 560064, Karnataka, India Ashoka Trust for Research in Ecology and the Environment Bengaluru India; 2 Entomology and Nematology Department, University of Florida, 1881 Natural Area Drive, Gainesville, FL, 32611, USA University of Florida Gainesville United States of America

**Keywords:** Abor Expedition, Arunachal Pradesh, aspirator, East Siang, taxonomy

## Abstract

A new ant species, *Paraparatrechinaneela***sp. nov.**, with a captivating metallic-blue color is described based on the worker caste from the East Siang district of Arunachal Pradesh, northeastern India. This discovery signifies the first new species of *Paraparatrechina* in 121 years, since the description of the sole previously known species, *P.aseta* (Forel, 1902), in the Indian subcontinent.

## ﻿Introduction

The formicine ant genus *Paraparatrechina* was originally described by Donisthorpe (1947) as a subgenus of *Paratrechina* Motschoulsky, 1863, with Pa. (Paraparatrechina) pallida (Donisthorpe, 1947) as type species by monotypy. Later, Brown (1973) treated *Paraparatrechina* as a provisional junior synonym of *Paratrechina*, and Trager (1984) confirmed this synonymy, citing the lack of monophyly of the subgenus based on a morphological assessment. However, LaPolla et al. (2010a) redefined *Paratrechina* as a monotypic genus with *Pa.longicornis* (Latreille, 1802) based on a phylogenetic analysis of the *Prenolepis* genus-group. That study also recovered *Paraparatrechina* as a valid monophyletic genus, distinguishable from its sister taxa by the uniform, erect setal pattern on the mesosoma. Currently, the *Prenolepis* genus-group comprises the genera *Euprenolepis* Emery, 1906, *Nylanderia* Emery, 1906, *Paraparatrechina*, *Paratrechina*, *Prenolepis* Mayr, 1861, and *Pseudolasius* Emery, 1887 (LaPolla et al. 2010a).

*Paraparatrechina* are generally small ants, measuring 1–2 mm long, and they are typically found in the Afrotropical, Australasian, Indomalayan, Oceanian, and Palearctic biogeographic regions (LaPolla et al. 2010b; AntWeb 2023a). The genus can be easily distinguished from other formicine ant genera by a unique mesosomal setal pattern, which includes two pairs of erect pronotal setae, one pair of mesonotal setae, and one pair of propodeal setae (LaPolla et al. 2010a). The genus is often confused with *Nylanderia*. However, *Nylanderia* lacks a pair of erect propodeal setae and has six mandibular teeth instead of five (LaPolla et al. 2010b).

*Paraparatrechina* is present in various tropical environments, ranging from rainforests to forest clearings, and can be found in a wide spectrum of habitats, ranging from leaf litter on the ground to high up in the canopy (LaPolla et al. 2010b). Afrotropical species, for example *P.weissi* (Santschi, 1910) and *P.bufona* (Wheeler, 1922), are the only known polymorphic species of *Paraparatrechina*, displaying several morphological characteristics indicative of a hypogaeic lifestyle (LaPolla et al. 2010a).

Currently, *Paraparatrechina* encompasses 38 valid species and four valid subspecies (Bolton 2023). The Indomalayan biogeographic region has 14 known species, while *P.aseta* (Forel, 1902) is the only known species in the Indian subcontinent until now (Bharti and Wachkoo 2014). This species has been reported in several states of India, including Gujarat, Himachal Pradesh, Jammu and Kashmir, Sikkim, Nagaland, and West Bengal (Bharti et al. 2016; Janicki et al. 2016; Guénard et al. 2017). In this study, we describe and illustrate *P.neela* sp. nov., which was discovered in the foothills of the Eastern Himalayas of India. This find comes 121 years after the discovery of the only previously known Indian species, *P.aseta*.

During the period of colonial rule in India, a scientific expedition to document the natural history and geography of the Siang Valley of the Eastern Himalayas accompanied a punitive military expedition against the indigenous people there in 1911–12 (Army Intelligence Branch 1911). Originally known as the Abor Expedition, the findings of the expedition were published in several volumes from 1912 to 1922 in the *Records of the Indian Museum*. Now, a century later, a team of researchers has been engaged in a series of expeditions under the banner “Siang Expedition”, funded by the National Geographic Society through the wildlife-conservation expedition grant (NGS-71945c-20), to resurvey the biodiversity of the region. In May 2022, among several other ant species from various genera, we collected two worker specimens of *P.neela* sp. nov. from Yingku village, in East Siang District of Arunachal Pradesh, northeastern India. This remarkable new species represents the first documented occurrence of the genus in Arunachal Pradesh and only the second *Paraparatrechina* species known from the Indian subcontinent.

## ﻿Materials and methods

**Figure 1.**
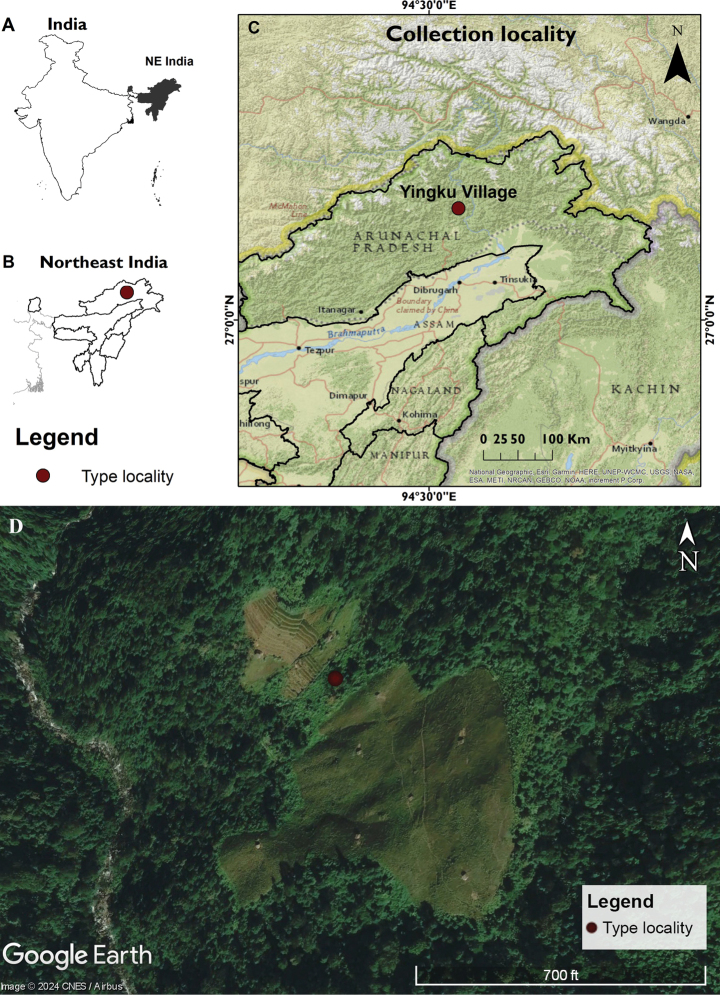
Map showing the type locality of *P.neela* sp. nov. in the Yingku Village, Arunachal Pradesh, northeastern India **A** India, with the North East Region (NER) shown in gray **B** states of the NER with type locality in Arunachal Pradesh **C** Arunachal Pradesh showing the location of the type locality (Yingku village) **D** Google Earth satellite image showing the type locality (Source: Google Earth Pro 2023, accessed on 11 February 2024).

Two worker specimens belonging to *Paraparatrechina* were collected from a secondary forest at an elevation of 803 m in Yingku village, which is located in East Siang District of Arunachal Pradesh, northeastern India (Fig. 1). East Siang District is encompassed between latitudes 27°43'N to 29°20'N and longitudes 94°42'E to 95°35'E and has an area of 4005 km^2^. It has tropical and humid-subtropical climate, with temperatures of 18–28 °C and an average annual rainfall of 4168 mm (Yumnam et al. 2013; Yogesh Kumar et al. 2022). These specimens were collected from debris in a hole in a tree trunk 3 m above the ground. We used an aspirator to extract the specimens and preserved them in absolute alcohol. The specimens were point mounted and examined under a Zeiss SteREO Discover.V8 microscope.

**Figure 2.**
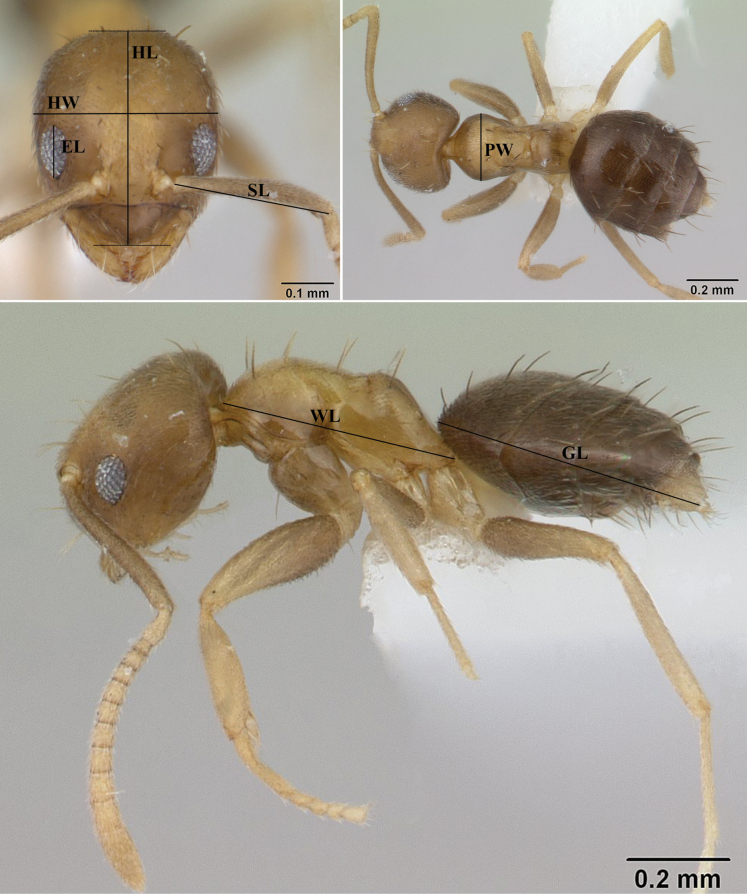
*Paraparatrechinaumbranatis* LaPolla & Cheng, 2010, showing schematic representation of the body measurements. Abbreviations are defined in materials and methods. Photo credit: April Nobile, www.antweb.org, CASENT0178764 (AntWeb 2024).

The identifications of the specimens were made by referring to available taxonomic keys by LaPolla et al. (2010a, 2010b) and comparing them with images of the types of all known *Paraparatrechina* species, except for *P.bufona*, *P.kongming* (Terayama, 2009), *P.nana* (Santschi, 1928), *P.sakuya* Terayama, 2013, *P.sordida* (Santschi, 1914), and *P.tapinomoides* (Forel, 1905), which are not available on AntWeb (2023a). We checked original descriptions and available illustrations for specimens that did not have type images. The unique metallic-blue coloration of the body, in combination with the head shape, sculpture and pubescence patterns, helped us to confirm the status of new species. Stacked focus montage images of the new species were captured at 200× magnification using a Keyence VHX 6000 digital microscope. Final figures were prepared using Adobe Photoshop v. 25. The map was prepared using ArcGIS v. 10.4.1 (ArcGIS 2023). The holotype and paratype specimens are deposited in ATREE Insect Museum, Bangalore, India (**AIMB**). Body measurements are in millimeters and were taken with AxioVision v. 4.8 software (Carl Zeiss, Germany) and recorded with two decimal places. Body measurements and indices (Fig. 2) follow LaPolla and Fisher (2014).

Eye length (EL): maximum length of compound eye in full-face view.

Head length (HL): the length of the head proper, excluding the mandibles;

measured in full-face view from the midpoint of the anterior clypeal margin to a line drawn across the posterior margin from its highest points.

Head width (HW): the maximum width of the head in full-face view.

Scape length (SL): the maximum length of the antennal scape excluding the condylar bulb.

Pronotal width (PW): the maximum width of the pronotum in dorsal view.

Weber’s length (WL): in lateral view, the distance from the posteriormost border of the metapleural lobe to the anteriormost border of the pronotum, excluding the neck.

Gaster length (GL): the length of the gaster in lateral view from the anteriormost point of the first gastral segment (third abdominal segment) to the posteriormost point.

Total length (TL): HL + WL + GL.

Cephalic index (CI): (HW / HL) × 100.

Relative eye length index (REL): (EL / HL) × 100.

Scape index (SI): (SL / HW) × 100.

## ﻿Results

### ﻿ Paraparatrechina

Taxon classificationAnimaliaHymenopteraFormicidae

Donisthorpe, 1947

EC287B7E-299E-5D5D-B527-75382F5F0BBC

Paraparatrechina Donisthorpe 1947: 192, as a subgenus of Paratrechina. Type species: Paratrechinapallida, by monotypy.
Paraparatrechina as junior synonym of Paratrechina: Brown 1973: 183; Trager 1984: 58.

#### Status as genus.

LaPolla et al. 2010a: 128.

#### Diagnosis.

***Worker*** (adapted from LaPolla et al. 2010a): *Paraparatrechina* workers can be identified by the following combination of characters: antenna with 12 segments; mandible with 5 or 6 teeth; maxillary palp and labial palp 6- and 4-segmented, respectively; erect setae on head form a distinct pattern consisting of 4 setae along posterior margin and 6 or 7 rows of paired setae from posterior margin to clypeal margin; erect setae absent on antennal scapes and legs; head excluding clypeal surface and mesonotal dorsum with dense pubescence; eyes typically well developed and positioned laterally towards the midline of head; erect mesosomal setae distinctly paired–2 pairs on pronotum, 1 pair on mesonotum, and 1 pair on propodeum; propodeum dorsal face typically short compared to its longer posterior face; general overall mesosoma shape compact, although a few species have elongated mesosoma.

***Queen*** (adapted from Cantone 2018): antennae filiform with 12 segments, extending beyond occipital margin; antennal socket located near posterior edge of clypeus; forewings exhibit typology III, formica type, with a closed marginal cell; hindwings display typology II, lacking anal 2 vein; mandibles triangular and dentate; palp formula 6:4, or in *P.bufona* and *P.weissi*, maxillary palp consists of 5 segments; mesosomal setal pattern same as in workers; metatibiae with a single spur.

**Male** (adapted from Cantone 2017): antennae with 13 segments, with the scape extending beyond occipital margin; first funicular segment longer and wider than second; forewings exhibit typology III, with a closed marginal cell; hindwings correspond to typology II; mandibles edentate.

### ﻿Key to *Paraparatrechina* species of the Indian subcontinent based on the worker caste

We recognize the uncertainty in the taxonomy of Indomalayan *Paraparatrechina*, as some species do not have the typical characteristics of the genus, and for this reason, we have not provided a key to the Indomalayan species. A comprehensive revision is necessary before reliable taxonomic key to the Indomalayan *Paraparatrechina* can be made.

The Indian subcontinent has only two species of *Paraparatrechina*, *P.aseta* and *P.neela* sp. nov. See the worker description of *P.neela* for a detailed comparison with *P.aseta*.

**Table:** 

1	Body uniformly light brown; head subrectangular; mandible with 6 teeth in the masticatory margin	***P.aseta***
–	Body largely metallic blue; head subtriangular; mandible with 5 teeth in the masticatory margin	***P.neela* sp. nov.**

### ﻿ Paraparatrechina neela sp. nov.

Taxon classificationAnimaliaHymenopteraFormicidae

8E4EE5CC-4108-5974-B721-EA995326F867

https://zoobank.org/E1CB7812-6BF7-4CCC-A319-0D75A493416F

Figs 3 , 4

#### Material examined.

***Holotype***: worker, point mounted. Original label: “India: Arunachal Pradesh, East Siang District, Yingku Village, 28.4606°N, 94.8841°E, 803 m a.s.l., aspirator, 7 May 2022, Priyadarsanan DR leg.”; AIMB/Hy/Fr 25006. ***Paratype***: 1 worker; same data as holotype; AIMB/Hy/Fr 25007.

#### Worker description.

Measurements (in mm) and indices:

Holotype worker: EL 0.14; HL 0.50; HW 0.42; SL 0.51; PW 0.29; WL 0.53; GL 0.66; TL 1.69; CI 84; REL 28; SI 121.

Paratype worker: EL 0.13; HL 0.59; HW 0.43; SL 0.50; PW 0.28; WL 0.57; GL 0.66; TL 1.76; CI 72; REL 22; SI 116.

#### Diagnosis.

*Paraparatrechinaneela* sp. nov. has the following unique combination of characters: 1) body opaque and largely metallic blue, except antennae, mandibles, and legs; 2) total length < 2 mm; 3), eyes large relative to head length (REL > 22); 3) scape with appressed pubescence and scape surpasses posterior margin of head by approximately length of first 4 funicular segments; 4) propodeal dorsal face short and angular, with a long declivitous face.

**Figure 3.**
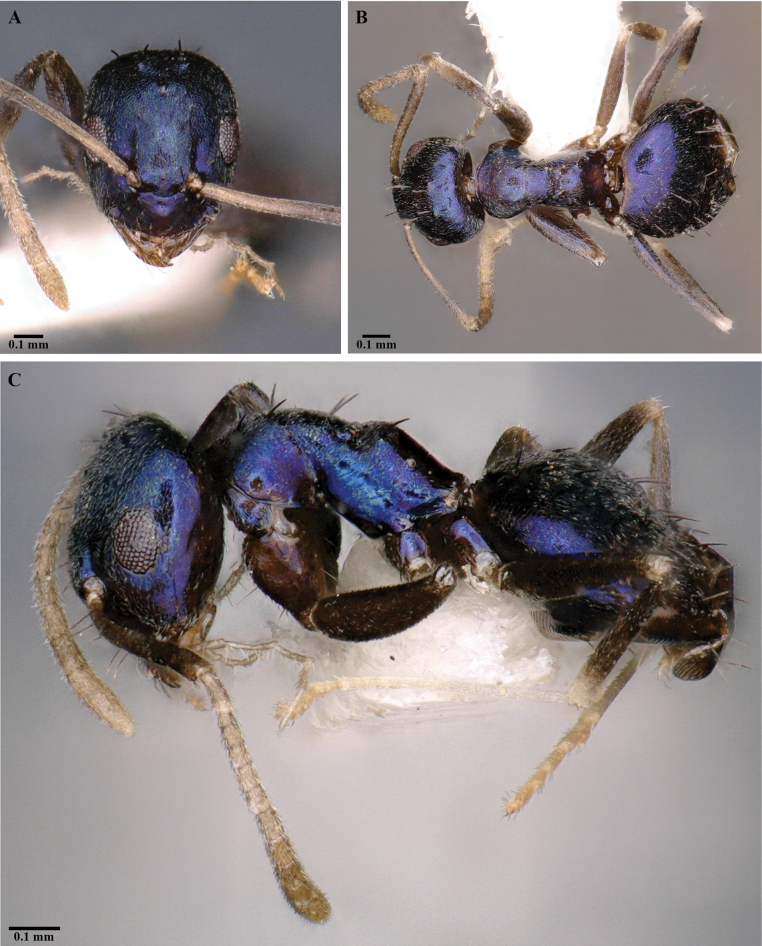
*Paraparatrechinaneela* sp. nov., holotype worker AIMB/Hy/Fr 25006 **A** head in full-face view **B** body in dorsal view **C** body in profile view.

**Figure 4.**
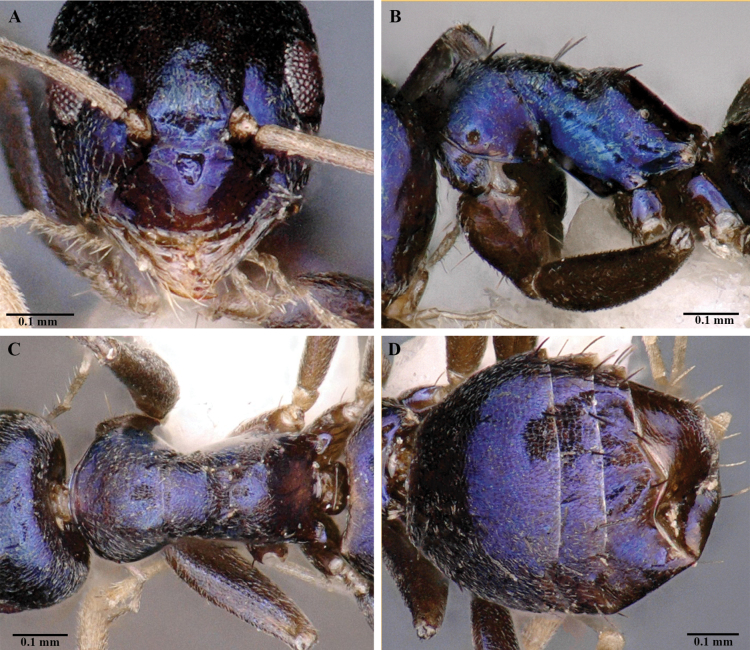
*Paraparatrechinaneela* sp. nov., holotype worker AIMB/Hy/Fr 25006 **A** clypeus and mandibles **B** mesosoma in profile view **C** mesosoma in dorsal view **D** gaster in dorsal view.

***Head.*** In full-face view (Fig. 3A), subtriangular, 1.2× longer than wide; posterior margin of head convex. Mandible triangular, masticatory margin with 5 teeth (Fig. 4A), 1 long apical tooth followed by acutely triangular tooth, 2 minute denticles and a triangular basal tooth: maxillary palp and labial palp with palp formula, PF (6:4). Antennae with 12 segments; scape surpasses posterior margin of head approximately by the length of first 4 funicular segments. In profile view, clypeal disc projects, medially carinate. In full-face view, anterior clypeal margin convex. Eyes large, REL 22–28, oval; ocelli present, only median ocellus visible, other two ocelli relatively concealed, indistinct (Fig. 3A).

***Mesosoma.*** Compact in lateral view, pronotum convex with short dorsal face in lateral view (Figs 3C, 4B). Mesonotum sloping towards metanotum, propleura and mesopleura demarcated by a distinct promesonotal suture; mesopleura and metapleura demarcated by indistinct metanotal groove; propodeum raised, propodeal dorsal face short, angular, with a long declivitous face; propodeal spiracle distinct (Fig. 3C).

***Metasoma.*** Petiole length 0.05 mm, strongly compressed antero-posteriorly. Gaster with 5 tergites, anterior margin concave and forming sharp edges in dorsal view (Figs 3B, 4D). Acidopore distinct apically (Fig. 3C).

***Sculpture.*** Body covered with fine punctures; mandibles with longitudinal striations (Fig. 4A); clypeus, mesopleura, and propodeal declivity smooth and shiny (Fig. 4A–C).

***Pilosity.*** Short, decumbent pubescence covers most of the body. Distinctly paired dark setae present from anterior clypeal margin to propodeum; 8 pairs on head from posterior region to clypeus; 2 pairs on pronotum, 2 pairs on mesonotum, 1 pair on propodeum (Figs 3C, 4B). Setae shorter on head posterior to eyes and gaster and longer on anterior of head and mesosoma.

***Color*.** Body largely iridescent blue, with a purple tinge and white pubescence; legs and antennae brown at base, dark to yellowish brown at middle, white at the tip; mandible yellowish brown. Gaster blue in anterior region, brown towards posterior end.

#### Etymology.

The specific epithet *neela* is a noun in apposition, signifying the color blue in most Indian languages. It is used to describe the unique blue or sapphire color of this species.

#### Species comparison.

**Figure 5.**
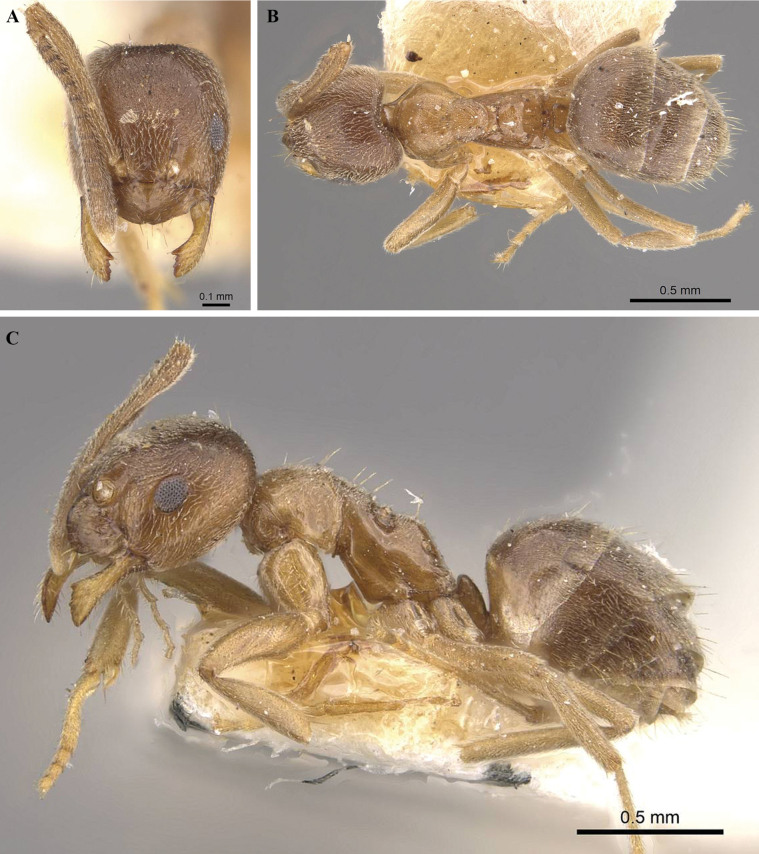
*Paraparatrechinaaseta*, syntype worker **A** head in full-face view **B** body in dorsal view **C** body in profile view. Photo credit: Will Ericson, www.antweb.org, CASENT0910999 (AntWeb 2023b).

*Paraparatrechinaneela* sp. nov. is easily distinguishable from all known species of *Paraparatrechina* by its metallic-blue body. It can be separated from *P.aseta*, the only other known species from the Indian subcontinent (Fig. 5A–C) by the following characteristics: 1) body largely metallic blue, except antennae, mandibles, and legs in *P.neela* (body uniformly light brown in *P.aseta*); 2) in full-face view, head subtriangular with strongly convex lateral margin in *P.neela* (head subrectangular with gently convex lateral margin in *P.aseta*); 3) anterior clypeal margin convex in *P.neela* (anterior clypeal margin medially concave in *P.aseta*); 4) mandible with five teeth in the masticatory margin in *P.neela* (mandible with six teeth in *P.aseta*); 5) propodeal dorsal face in lateral view raised in *P.neela* (propodeal dorsal margin flat and continuous with rest of mesosoma in *P.aseta*). *Paraparatrechinaneela* is similar to *P.buttelibryanti* (Forel, 1916), another Indomalayan species (Wheeler 1919), in body size, eye length, antennal scape surpassing occipital margin, and a raised propodeal dorsal face with a long declivitous face. However, *P.neela* can be easily separated from *P.buttelibryanti* by the following characteristics: 1) body largely metallic blue in *P.neela* (body castaneous brown; head, thorax, and gaster with metallic reflections in *P.buttelibryanti*); 2) legs with thick appressed pubescence in *P.neela* (legs with sparse pubescence in *P.buttelibryanti*); 3) overall body opaque with fine punctures in *P.neela* (thorax and gaster distinctly shagreened in *P.buttelibryanti*); 4) head subtriangular, longer than wide in *P.neela* (head subrectangular, as long as wide in *P.buttelibryanti*).

## ﻿Discussion

*Paraparatrechina* is a relatively underexplored genus but with an expected species diversity much higher than what is currently known (LaPolla et al. 2010b; LaPolla and Fisher 2014). Previous studies indicate that the *Prenolepis* genus-group, which includes *Paraparatrechina*, originated and diversified during the late Paleocene and Eocene, between 45 and 60 mya (Blaimer et al. 2015; Matos-Maraví et al. 2018). Blaimer et al. (2015) estimated ancestral crown age of the genus ranges from 26.8 to 31.4 Ma. Similarly, the estimated crown age of a clade within *Paraparatrechina* is 23.5 Ma (Matos-Maraví et al. 2018). Matos-Maraví et al. (2018) suggested that the *Prenolepis* genus-group most likely originated in continental Southeast Asia. It points to the possibility of dispersal and colonization of this group from Southeast Asia to India. Further explorations are imperative to unravel the influence of the Himalayas and the Western Ghats on this group’s evolution and dispersal.

*Paraparatrechina* species are typically found at elevations below 800 m, although a few inhabit elevations around 1500 m (AntWeb 2023a). However, a few species, such as *P.minutula* (Forel, 1901) and *P.kongming* (Terayama, 2009), are known to occupy higher elevations above 2000 m (2300 m and 2500 m, respectively). *Paraparatrechinaneela* sp. nov. was collected from an elevation of 803 m. This species showcases a unique metallic blue coloration not observed in any other species within this genus. However, some *Paraparatrechina* species do exhibit color reflections or iridescence, like *P.iridescens* (Donisthorpe, 1942).

Blue coloration in animals, except in marine sponges, is a relatively rare phenomenon. However, there are several blue species of vertebrates, like fish, frogs, and birds, as well as invertebrates, such as spiders (Bagnara et al. 2007; Doucet and Meadows 2009; Umbers 2013; Chomphuphuang et al. 2023). While blue coloration is common among many insects, particularly in hymenopterans such as Apoidea, Chrysididae and Ichneumonidae, it is very rare in Formicidae. Blue colour in insects is usually produced by the arrangement of biological photonic nanostructures rather than pigments, and it has independently evolved in various groups (Prum 1999; Seago et al. 2009; Hsiung et al. 2015; Chomphuphuang et al. 2023). This vibrant feature raises intriguing questions. Does it help in communication, camouflage, or other ecological interactions? Delving into the evolution of this conspicuous coloration and its connections to elevation and the biology of *P.neela* presents an exciting avenue for research.

## Supplementary Material

XML Treatment for Paraparatrechina

XML Treatment for Paraparatrechina neela

## ﻿Additional information

### Author ORCIDs

Ramakrishnaiah Sahanashree https://orcid.org/0000-0002-3040-2102

Aswaj Punnath https://orcid.org/0000-0001-8034-6578

Dharma Rajan Priyadarsanan https://orcid.org/0000-0001-8137-3404

### Data availability

All of the data that support the findings of this study are available in the main text.
